# Novel anti-cancer drug COTI-2 synergizes with therapeutic agents and does not induce resistance or exhibit cross-resistance in human cancer cell lines

**DOI:** 10.1371/journal.pone.0191766

**Published:** 2018-01-24

**Authors:** Saman Maleki Vareki, Kowthar Y. Salim, Wayne R. Danter, James Koropatnick

**Affiliations:** 1 Cancer Research Laboratory Program, Lawson Health Research Institute, London, Ontario, Canada; 2 Cotinga Pharmaceuticals (formerly Critical Outcome Technologies Inc.), London, Ontario, Canada; 3 Department of Microbiology and Immunology, Western University, London, Ontario, Canada; 4 Department of Pathology, Western University, London, Ontario, Canada; 5 Department of Oncology, Western University, London, Ontario, Canada; 6 Department of Physiology and Pharmacology, Western University, London, Ontario, Canada; Columbia University, UNITED STATES

## Abstract

Emerging drug-resistance and drug-associated toxicities are two major factors limiting successful cancer therapy. Combinations of chemotherapeutic drugs have been used in the clinic to improve patient outcome. However, cancer cells can acquire resistance to drugs, alone or in combination. Resistant tumors can also exhibit cross-resistance to other chemotherapeutic agents, resulting in sub-optimal treatment and/or treatment failure. Therefore, developing novel oncology drugs that induce no or little acquired resistance and with a favorable safety profile is essential. We show here that combining COTI-2, a novel clinical stage agent, with multiple chemotherapeutic and targeted agents enhances the activity of these drugs *in vitro* and *in vivo*. Importantly, no overt toxicity was observed in the combination treatment groups *in vivo*. Furthermore, unlike the tested chemotherapeutic drugs, cancer cells did not develop resistance to COTI-2. Finally, some chemo-resistant tumor cell lines only showed mild cross-resistance to COTI-2 while most remained sensitive to it.

## Introduction

Emergence of drug-resistant clones and treatment-associated toxicities are two major barriers to successful cancer treatment [1]. Drug resistance can result in rapid disease progression either during or shortly after completion of treatment. Cytotoxic anticancer drugs designed to inhibit growth of fast-dividing tumor cells are often not target-specific and are limited by their off-target toxicities. Precision medicine drugs are designed to be target-specific and to induce fewer undesirable side effects than conventional chemotherapy. Nevertheless, cancer cells often develop resistance to targeted agents [2]. Combining two or more agents with unrelated mechanisms of action and different modes of drug resistance is often used as a strategy to minimize drug resistance in cancer [3]. Although, combination treatments can increase response rates and reduce resistance, they are limited by treatment-associated side effects and a narrow safety window [4]. Therefore, development of new drugs less susceptible to known resistance mechanisms can improve anti-tumor activity [5] and the use of such drugs to which cancer cells cannot easily develop cross-resistance is crucial to the success of new drug combinations.

COTI-2, a novel small molecule currently in a phase I clinical study of gynaecological malignancies and head and neck squamous cell carcinoma (HNSCC), was designed using CHEMSAS^®^, a proprietary computational platform. We recently reported that as a single agent COTI-2 exhibits potent anti-proliferative activity against a wide variety of human cancer cell lines *in vitro* (at nanomolar concentrations) and against human tumor xenografts *in vivo* [6]. Although the precise mechanism of action of COTI-2 remains to be determined, this agent was not a traditional kinase inhibitor nor did it inhibit the ATPase activity of Hsp90 [6].

In this study, COTI-2 was evaluated in combination with cytotoxic chemotherapeutics (platinum-containing agents, taxanes, *vinca* alkaloids, and antimetabolites) and targeted agents (mTOR and EGFR inhibitors) to determine whether COTI-2 would enhance their activity. COTI-2 was synergistic in multiple combinations without exerting significant toxicities *in vivo*. COTI-2 was also evaluated for its ability to induce acquired resistance in human cancer cells following multiple rounds of exposure. Unlike common chemotherapeutic drugs such as paclitaxel and cisplatin, cancer cells did not develop acquired resistance to COTI-2. Finally, tumor cells resistant to chemotherapeutic agents (cisplatin, paclitaxel, and 5-FUdR) exhibited no or little cross-resistance to COTI-2. These data cumulatively suggest that COTI-2 may have potential in salvage treatment after first- and second-line treatment failure with common therapies.

## Materials and methods

### Cell lines

All cell lines, except the head and neck squamous cell carcinoma (HNSCC) HN-5a, were obtained from the American Type Culture Collection (ATCC) in the last 5 years and maintained under standard conditions (37°C, 5% CO_2_) in αMEM or DMEM medium (Wisent Inc., Mississauga, Canada) supplemented with 10% FBS without antibiotics. The HN-5a cell line was a gift from Dr. Henry Lapointe in 1990 [7]. Human AN3-CA endometrial adenocarcinoma cells were maintained in RPMI 1640 (Hyclone, Logan, UT) supplemented with 5% FBS and 10% CO_2_. All cell lines were authenticated using the GenePrint® 10 System (10 markers) by The Centre for Applied Genomics, The Hospital for Sick Children, Toronto, Canada.

### Drugs

COTI-2, supplied by Cotinga Pharmaceuticals (London, Ontario), was dissolved in 100% dimethyl sulfoxide (DMSO) stock solution and diluted in αMEM or DMEM plus 10% FBS so that final DMSO concentrations were 0.5–1.0%. For the AN3-CA xenograft studies, 235 mg of COTI-2 was mixed with 4.84 g of Captisol® (CydexPharmaceuticals, Lenexa, KS) in 10 ml of sterile water (Hospira, Inc., Lake Forest, IL). For the PANC-1 xenograft studies, COTI-2 was dissolved in phosphate-citrate buffer at pH 2.3.

Cisplatin, 5-FUdR (Sigma-Aldrich, St. Louis, MO), vinorelbine (GlaxoSmithKline, Brentford, UK), temsirolimus, rapamycin (Pfizer, New York, NY), gemcitabine (Eli Lilly, Indianapolis, IN), paclitaxel, carboplatin (Bristol-Myers Squibb Co., New York, NY), and vincristine were obtained from the London Health Sciences Centre Pharmacy (London Regional Cancer Program, London, Ontario). Cetuximab was purchased from ImClone Systems (New York, NY). Erlotinib was obtained from LC Laboratories (Woburn, MA).

### Proliferation assay

Tumor cell proliferation after drug treatment was measured as described previously [6]. Briefly, cancer cells were cultured overnight then allowed to proliferate in the presence of treatment drug then trypsinized and counted using a Beckman Coulter Z1 Particle Counter (Beckman, Mississauga, Ontario). AlamarBlue (ThermoFisher Scientific, Waltham, MA) was added to the treated cells after 4–7 days of drug exposure (approximately 4 doublings of untreated control cells) and cellular metabolic activity measured using a Wallac Victor2 multi-label counter (PerkinElmer, Gaithersburg, MD).

To assess additive, greater-than-additive (synergistic), or less-than-additive (antagonistic) effects in response to combination treatments, cells were treated with a concentration of COTI-2 that reduced proliferation by 25% in combination with increasing concentrations of the non-COTI-2 drug. Cell proliferation was calculated as a fraction of proliferation of cells treated with COTI-2 alone [8].

### Acquired resistance studies

Cancer cells were exposed to 4 rounds of selection that consisted of the addition of test agent to cells in medium plus 10% FBS for 4 days such that overall cell proliferation, assessed by alamarBlue and/or cell counting, was reduced by 50% (IC_50_ dose). Subsequently, the drug-containing medium was replaced with fresh complete medium without drug followed by a recovery period of 5 days. Naive tumor cells were exposed to the pre-determined IC_50_ dose of each test agent. The surviving 50% of cells from the initial IC_50_ test were harvested and cultured for 5 days, after which the new generation of cells was re-treated with the same agent and a new, higher IC_50_ value established (indicating increased drug resistance). The IC_50_ used to treat the cells of each subsequent generation was the IC_50_ measured after the immediately preceding round of selection. The procedure was repeated for 5 generations. Emerging resistance was identified as increasing IC_50_ values in successive generations.

### Cross-resistance studies

Human tumor cell lines with established resistance to chemotherapy drugs were evaluated for cross-resistance to COTI-2. Cell lines resistant to the various agents were selected and maintained as described previously [9]. The IC_50_ values were determined by measuring the proliferation of tumor cell growth. The alamarBlue assay was used to measure cellular metabolic activity after 4–7 days of drug exposure. The IC_50_ values were derived by interpolation of plotted data (mean values from 3 independent experiments ±SEM).

### *In vivo* xenograft studies

Human AN3-CA endometrial tumor cells (1 x 10^7^) were injected subcutaneously (SC) into the right flanks of 5-week-old female athymic nude mice, which consisted of 4 groups of 10 mice each. Body and tumor weights, measured as described previously [10], were recorded on the day of pair-matching and twice-weekly thereafter. COTI-2 (25 mg/kg) and vehicle control were administered intravenously (i.v.) 3 times weekly on alternate days until study end. Paclitaxel (5 mg/kg) was dosed daily for 5 days consecutively. The combination group received both paclitaxel (5 mg/kg) and COTI-2 (25 mg/kg) in a fashion identical to single agent treatment groups.

Human PANC-1 pancreatic tumor xenografts were established by injecting 2 x 10^6^ tumor cells per injection site into each flank of female NCr-*nu* mice (Taconic, Germantown, NY) then randomized into 6 groups of 12 mice each consisting of COTI-2 (125 mg/kg), gemcitabine (100 mg/kg), COTI-2 (125 mg/kg) plus gemcitabine (100 mg/kg), abraxane (15 mg/kg), COTI-2 (125 mg/kg) plus abraxane (15 mg/kg), or vehicle alone. COTI-2 was administered by oral gavage/*per os* (*p*.*o*.) with a treatment schedule of 5 days on and 2 days off per week. Gemcitabine was delivered intraperitoneally (i.p.) every second day for a total of 6 injections. Abraxane was administered i.v., once per day for 5 consecutive days. Dosing schedules for all treatment arms were identical except that COTI-2 treatment started one day after the first treatment with gemcitabine or abraxane. Tumor volumes, determined as described previously [6], were recorded multiple times during the course of the study.

All animal studies were approved by Western University’s Animal Use Subcommittee (approved protocol number 2008–069). Isoflurane was used for anesthesia and CO_2_ was used to euthanize the animals according to the institutional guidelines. Tumor growth inhibition (TGI) for both aforementioned animal studies was calculated according to the following formula:

$$TGI=\left[1-\frac{Xtreated(final)-Xtreated(day1)}{Xcontrol(final)-Xcontrol(day1)}\right]\;X\;100$$

### Statistical analysis

Student’s *t* test (2-tailed) was used to determine differences between two means. One-way ANOVA was used to assess differences among multiple means. A *p* value of 0.05 was selected *a priori* to indicate significant differences.

## Results

### Combining COTI-2 with paclitaxel and cisplatin enhances their activity in small cell lung cancer cells

**Fig 1:**
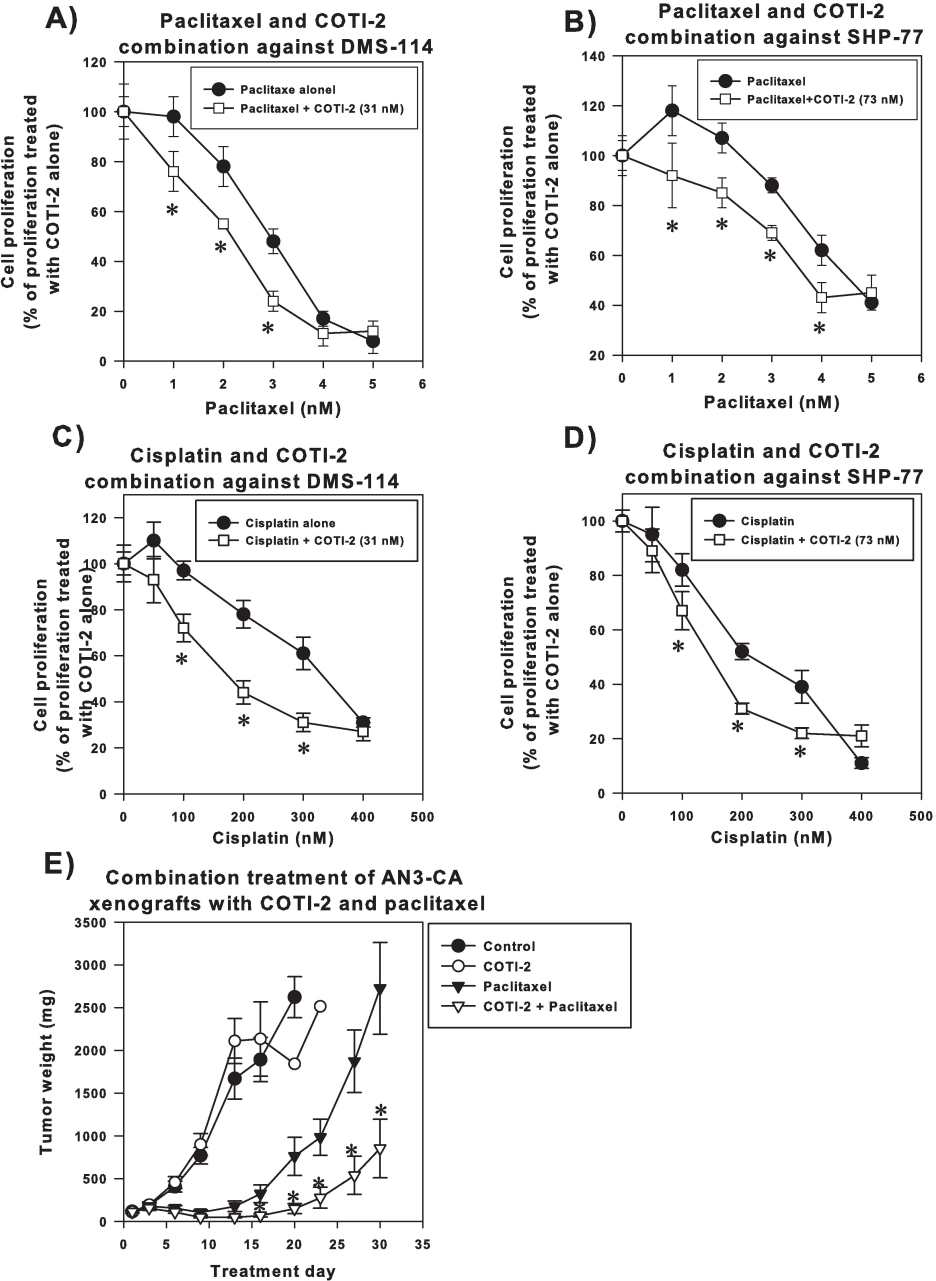
COTI-2 enhances the cytotoxic activity of paclitaxel and cisplatin. DMS-114 (A and C) and SHP-77 cells (B and D) were cultured overnight then exposed to the indicated doses of paclitaxel and cisplatin plus or minus a pre-determined dose of COTI-2 (IC_25_) for 4 days before cell viability was determined. The asterix (*) indicates a significant greater-than-additive effect in the combination therapy compared to single agent alone, *p*<0.05, Student’s *t*-test. Data are the average mean of 3 independent experiments ± SEM. (E) AN3-CA human endometrial cells (1 x 10^7^) were injected into the right flanks of athymic nude mice (n = 10 mice per group). Xenografts were grown to an average volume of 170 mm^3^ before animals received treatment i.v. Vehicle control and COTI-2 (25 mg/kg) were administered 3 times a week on alternate days until study end. The schedule for paclitaxel was daily for 5 days (5 mg/kg). In the combination arm, animals received COTI-2 (25 mg/kg) 3 times a week for the entire study and 5 injections of paclitaxel (5 mg/kg). *Significantly different from the paclitaxel alone treatment group, Student’s *t*-test, *p*<0.05. Error bars represent SEM.

Paclitaxel and cisplatin are commonly used as first-line chemotherapies in many cancers [11, 12], however, both exhibit dose-limiting toxicities and resistance [13, 14]. The combinations of COTI-2 plus paclitaxel as well as COTI-2 plus cisplatin enhanced the cytotoxic activity of both paclitaxel and cisplatin in SHP-77 and DMS-114 small cell lung cancer (SCLC) cells (Fig 1A–1D). These data suggest that COTI-2 can be used in combination with these first-line agents.

### COTI-2 induces significant tumor growth inhibition in combination with paclitaxel in the AN3-CA endometrial tumor model

COTI-2 was further evaluated in combination with paclitaxel in a human endometrial tumor model since paclitaxel is commonly used in the management of endometrial cancer [15]. Paclitaxel alone delayed the growth of human AN3-CA endometrial xenografts with a mean TGI of 74.1% on day 20 relative to the control group (Fig 1E). While COTI-2 treatment alone did not completely inhibit the growth of AN3-CA xenografts, the combination of paclitaxel and COTI-2 was more effective (TGI of 98.5% on day 20 relative to control group) than paclitaxel alone. In fact on day 30 (study end), the combination-treated group induced a significant TGI of 67.5% relative to the paclitaxel-treated group (Fig 1E). Furthermore, 4 of the 10 combination-treated animals exhibited complete tumor shrinkage by day 27. No significant weight difference was observed in mice treated with COTI-2 or paclitaxel compared to the control animals. Mice in the combination treatment arm initially lost 10% of their body weight, which was later recovered (S1 Fig). We have previously shown that COTI-2 induced apoptosis in tumor cells *in vitro* [6]. However, further studies are required to demonstrate a similar mechanism of action *in vivo*.

### COTI-2 increases the cytotoxic activity of carboplatin but not vinorelbine or gemcitabine

**Fig 2:**
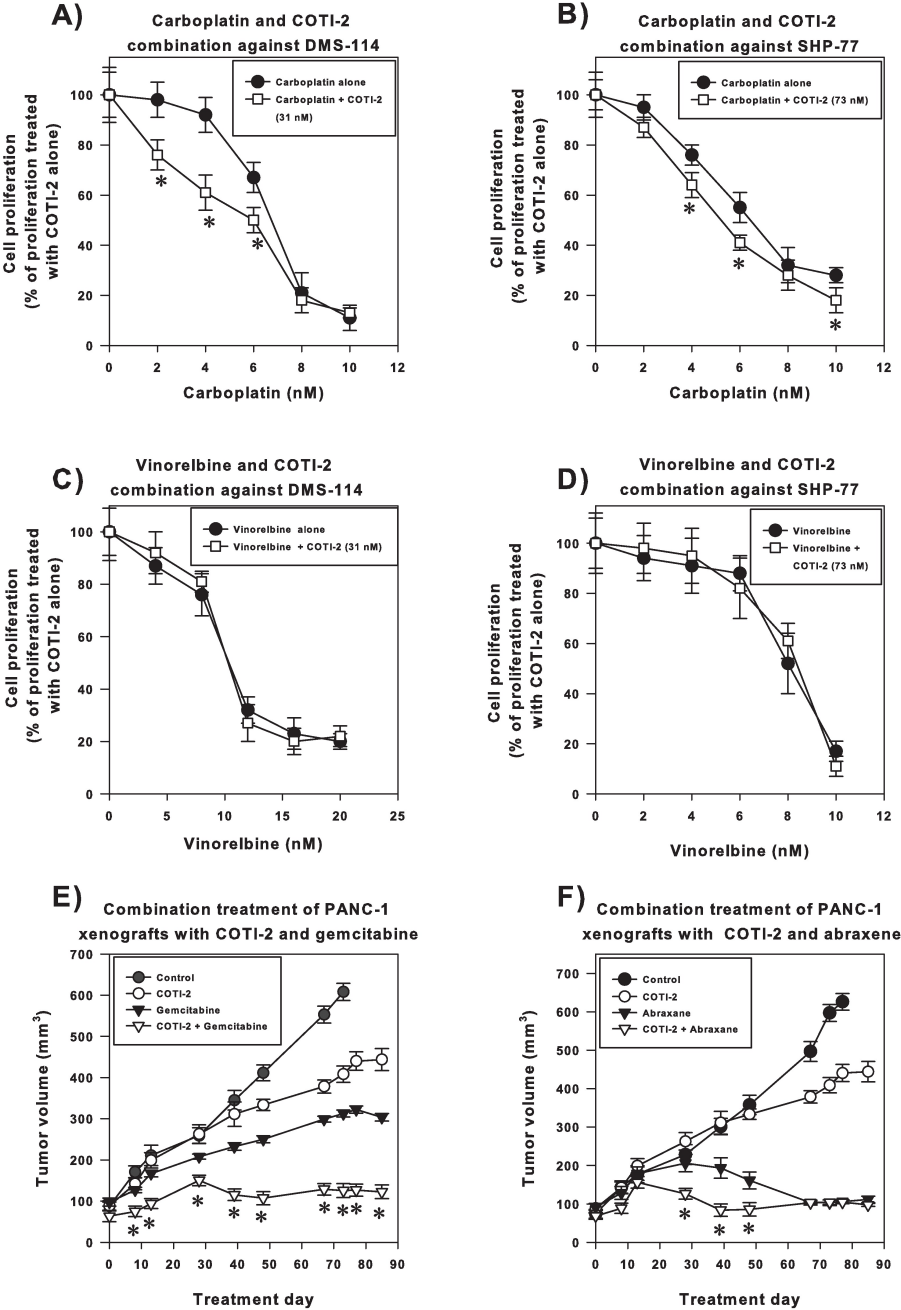
Only some chemotherapeutic drugs with similar molecular targets show enhanced activity when combined with COTI-2. DMS-114 (A and C) and SHP-77 (B and D) SCLC cells were treated with various concentrations of carboplatin (A and B) or vincristine (C and D) in combination with or without an IC_25_ concentration of COTI-2 for 4 days before cell viability was determined. The asterix (*) indicates a significant greater-than-additive effect in the combination therapy compared to single agent alone, *p*<0.05, Student’s *t*-test. Data are the average mean of 3 independent experiments ± SEM. (E and F) PANC-1 human pancreatic carcinoma cells (2 x 10^6^) were injected into each flank of NCr-*nu* mice (n = 12 mice per group). Xenografts were grown to ~100 mm^3^ before animals received treatment, which consisted of the vehicle control, COTI-2 (125 mg/kg), gemcitabine (100 mg/kg), or the combination (COTI-2 at 125 mg/kg and gemcitabine at 100 mg/kg) (E) or the vehicle control, COTI-2 (125 mg/kg), abraxane (15 mg/kg), or the combination (COTI-2 at 125 mg/kg and abraxane at 15 mg/kg) (F). COTI-2 was delivered *p*.*o*. with a schedule of 5 days on treatment and 2 days off weekly. Gemcitabine (100 mg/kg) was administered i.p., every second day, for a total of 6 injections. Abraxane (15 mg/kg) was administered i.v., once per day for 5 consecutive days. The dosing schedule for the combination treatments was identical to that of the single agent treatments for each drug. COTI-2 administration was initiated 1 day after treatment with either gemcitabine or abraxane. *Significantly different from single agent gemcitabine or abraxane treatment groups, Student’s *t*-test, *p*<0.05.

Similar to the cisplatin combination, COTI-2 in combination with carboplatin exhibited a synergistic effect in DMS-114 and SHP-77 SCLC cells (Fig 2A and 2B). COTI-2 in combination with carboplatin was also significantly more effective than carboplatin or COTI-2 treatment alone in inhibiting OVCAR-3 xenograft growth (S2 Fig). Moreover, combining COTI-2 with carboplatin at lower doses was safe for the animals while any doses of carboplatin at higher than 25 mg/kg in combination with COTI-2 or as single agent caused unacceptable toxicity in animals (S1 Table). Combining COTI-2 with vinorelbine in treatment of DMS-114 and SHP-77 SCLC cells *in vitro* did not enhance the activity of this drug (Fig 2C and 2D) nor did COTI-2 enhance the activity of gemcitabine (S3 Fig). We note that, while combining COTI-2 with vinorelbine (Fig 2C and 2D) or gemcitabine (S3 Fig) did not enhance the activity of those agents *in vitro*, COTI-2 did not antagonize the effect of these drugs.

### COTI-2 induces significant tumor growth inhibition in combination with gemcitabine and abraxane in the PANC-1 pancreatic tumor model

Gemcitabine and abraxane, which are mainly used for the treatment of pancreatic cancer [16], were evaluated in combination with COTI-2 in a PANC-1 human pancreatic tumor xenograft. Monotherapy with either COTI-2 or gemcitabine delayed growth of PANC-1 tumors in mice compared to vehicle control (Fig 2E and 2F). The combination of COTI-2 and gemcitabine, however, was more effective than either agent alone at inhibiting tumor growth (Fig 2E). In fact, the combination treatment induced significant tumor growth inhibition of 71.4% and 83.4% on day 85 relative to treatments with gemcitabine and COTI-2, respectively. The COTI-2 and abraxane combination reduced tumor volumes by 50–85% of those achieved by treatment with abraxane alone on days 28–48 (Fig 2F). However, the potent anti-tumor effect of abraxane masked, to some degree, the potential combination effect of abraxane and COTI-2.

### COTI-2 moderately increases the inhibitory effects of temsirolimus and rapamycin

**Fig 3:**
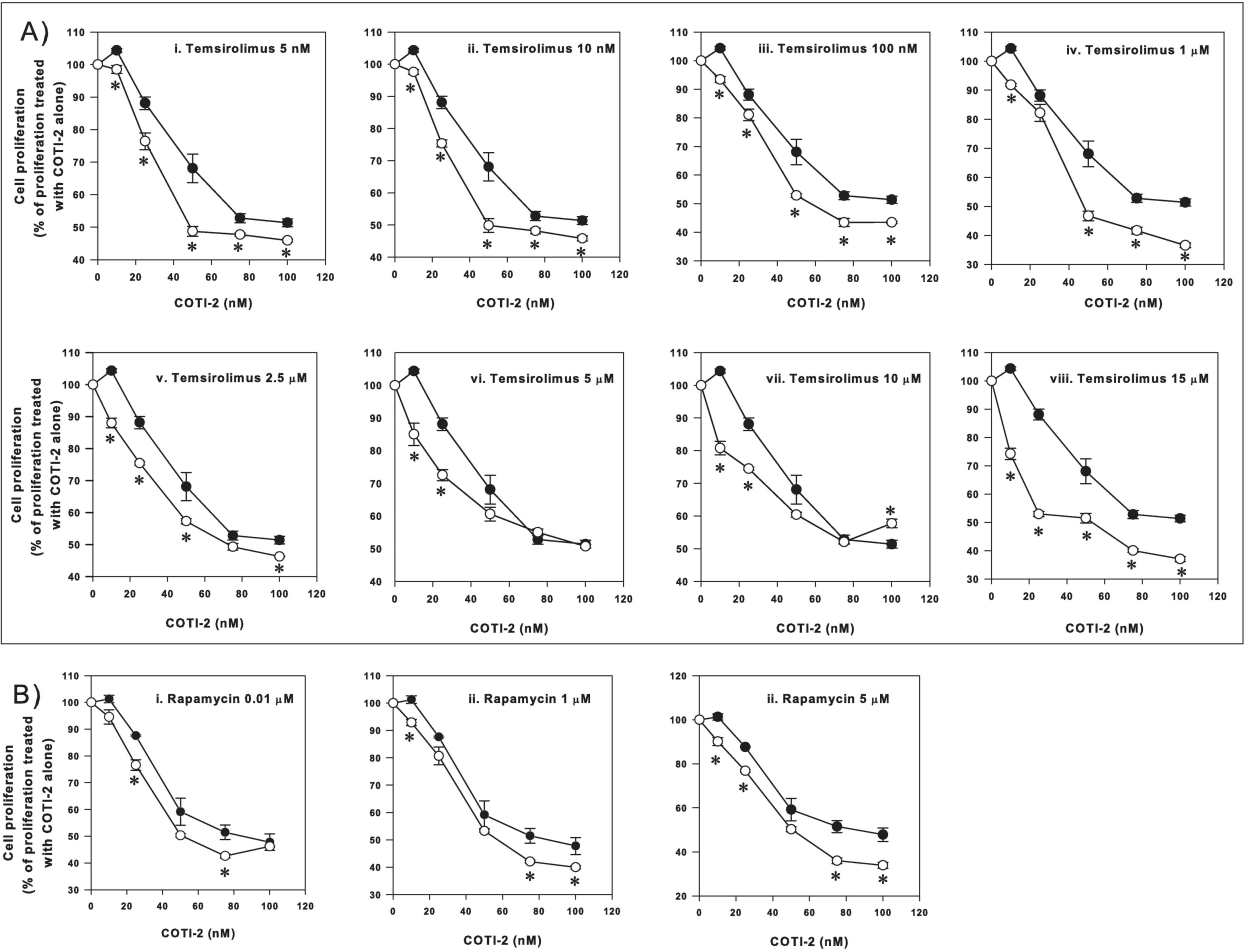
The effect of COTI-2 treatment on U87-MG cells in combination with temsirolimus and rapamycin. U87-MG human glioma cells were cultured in the presence of various concentrations of temsirolimus plus COTI-2 (A) or rapamycin plus COTI-2 (B) for 4 days before cell viability was determined. Black circles indicate the combination of COTI-2 and temsirolimus (A) or rapamycin (B) and the white circles indicate treatment with COTI-2 alone. Data are the average mean of 6 independent experiments ± SEM.*Significant difference, Student’s *t*-test, *p*<0.05.

Our preliminary mechanistic studies suggest that COTI-2 is a negative modulator of the PI3K/AKT/mTOR pathway [6, 17]. Therefore, we treated U87-MG glioblastoma cells with various concentrations of two mTOR inhibitors, temsirolimus and rapamycin, in combination with COTI-2. All lower (less than 60 nM) and some higher (up to 100 nM) concentrations of COTI-2 in combination with temsirolimus showed a synergistic effect (Fig 3A, panels [i] to [viii]). Synergism was less apparent at some higher concentrations of COTI-2 and temsirolimus. COTI-2 plus rapamycin effects were moderately synergistic for some of the lower concentrations of rapamycin (less than 5 μM) (Fig 3B). These data suggest that the optimal dose of each agent must be determined for any combination.

### Combination of COTI-2 with cetuximab and erlotinib synergistically enhances the activity of these targeted agents

**Fig 4:**
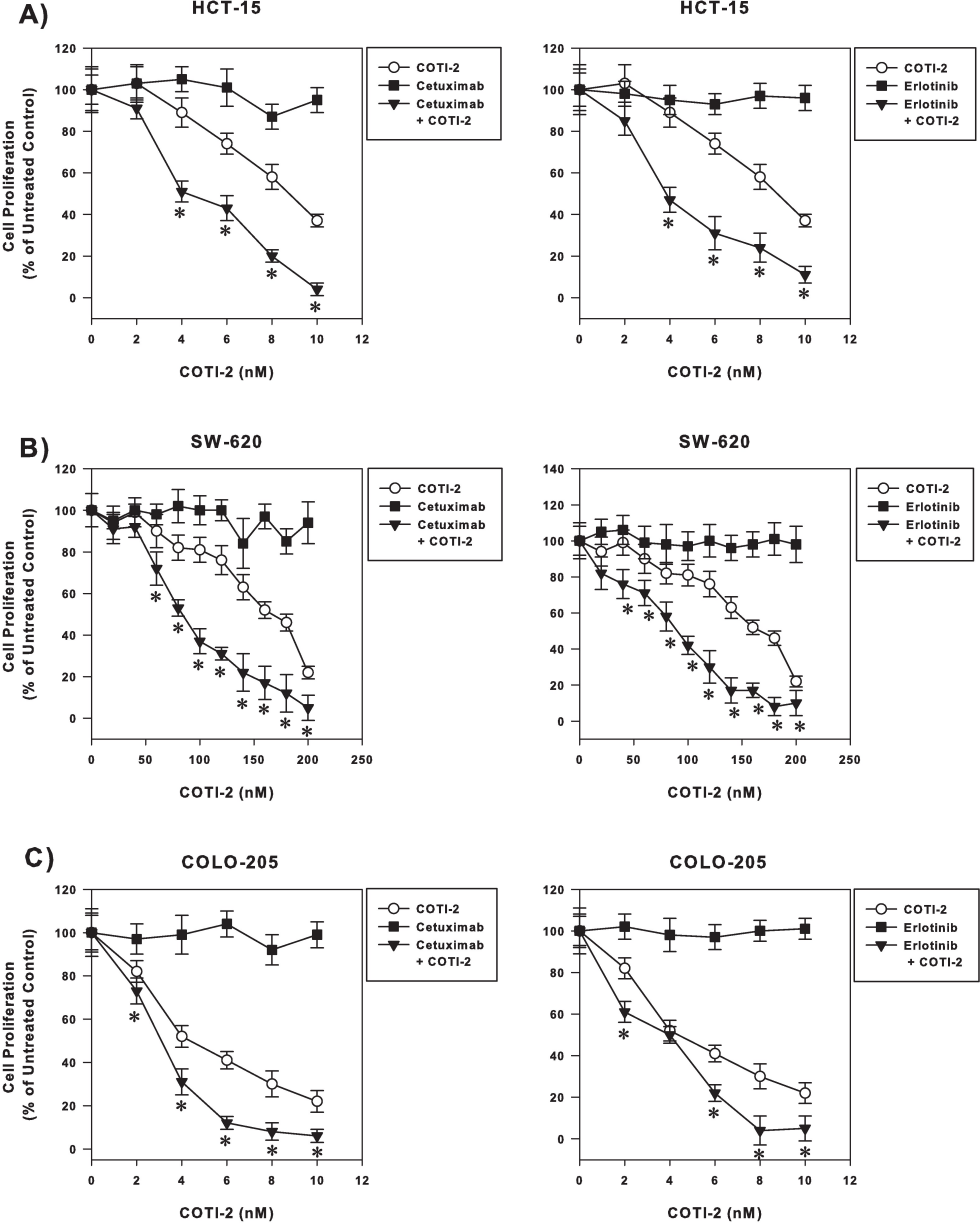
Combining COTI-2 with cetuximab and erlotinib synergistically enhances the efficacy of these drugs against human colorectal cancer cells. Human colorectal cancer cell lines HCT-15 (A), SW-620 (B), and COLO-205 (C) were treated with varying concentrations of COTI-2, cetuximab, erlotinib, or a combination of COTI-2 and either EGFR inhibitor. Tumor cells were allowed to proliferate for 4 days in the presence of drug(s) before cell viability was determined. All data points indicate the mean of 5 independent measures of viability ± SEM. *Significant difference from cells treated with COTI-2 alone using a Student’s *t*-test (*p*<0.05).

COTI-2 alone is more effective than cetuximab and erlotinib, two epidermal growth factor receptor (EGFR) targeting agents, at inhibiting the proliferation of multiple human colorectal cancer cell lines [6]. We examined whether combining COTI-2 with either agent would enhance their activity against human colorectal cancer cells, including HCT-15, SW-620, and COLO-205. Despite their lack of single agent activity, both cetuximab and erlotinib showed marked synergy when combined with COTI-2 (Fig 4). These data demonstrate that combining COTI-2 with cetuximab (anti-EGFR monoclonal antibody) or erlotinib (EGFR tyrosine kinase inhibitor) could enhance the activity of these agents regardless of their class activity.

### Cancer cells develop acquired resistance to paclitaxel and cisplatin but remain sensitive to COTI-2

**Fig 5:**
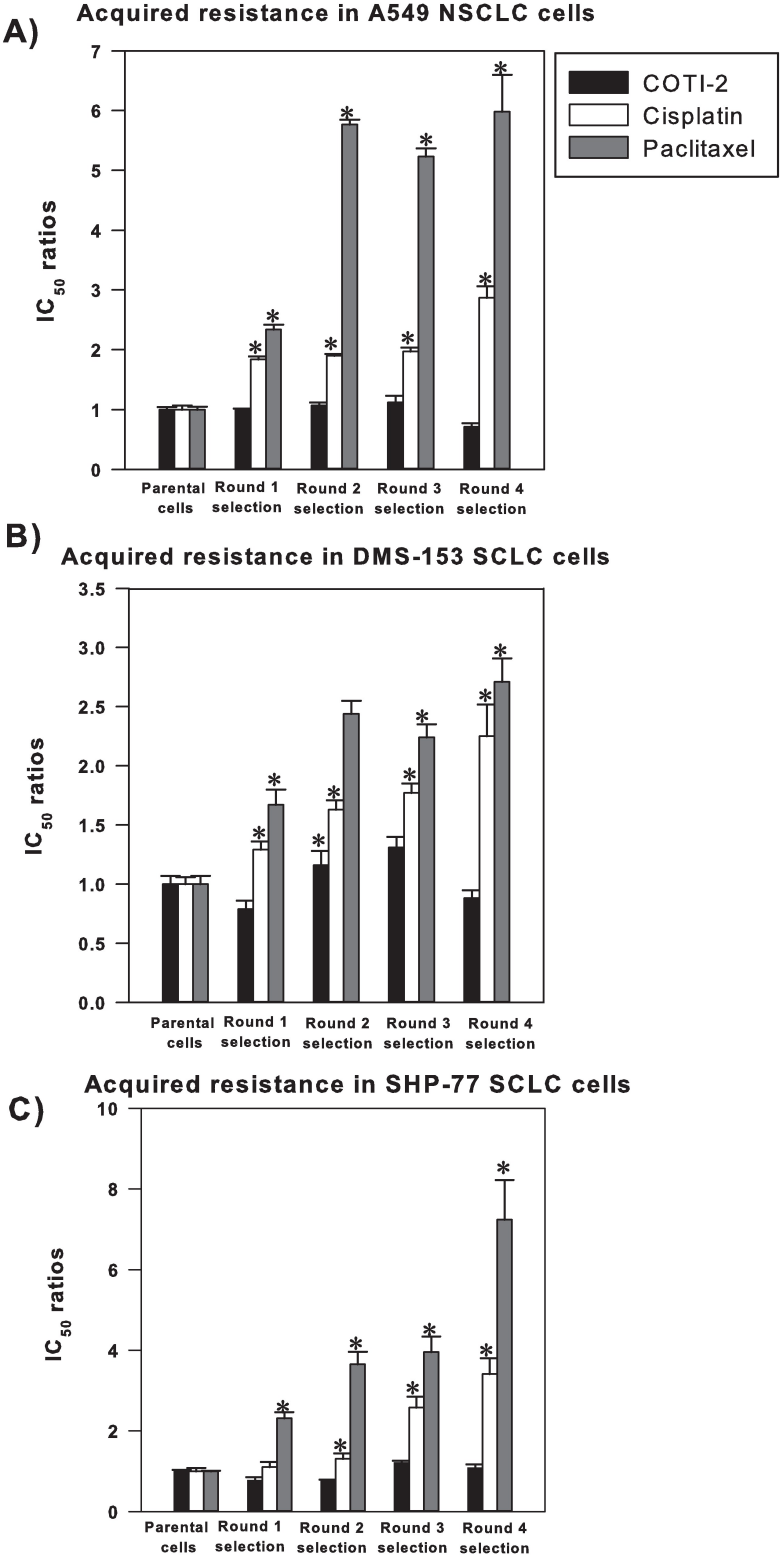
Cancer cells do not develop acquired resistance to COTI-2 unlike treatment with paclitaxel and cisplatin. A549 NSCLC (A), DMS-153 SCLC (B) and SHP-77 SCLC (C) cells were cultured in IC_50_ concentrations of COTI-2, paclitaxel, or cisplatin for 4 rounds of treatment (5 generations of cells including the parental cells). The surviving 50% of cells from the initial IC_50_ tested were harvested and cultured for 5 days, after which time this new generation of cells was re-treated with the same agent and a new IC_50_ value was established. Emerging resistance was identified by increasing IC_50_ values in successive generations. Significant differences were assessed by Student’s *t*-test (*p*<0.05). *Significantly different from parental cells treated with the mentioned drug. Data points indicate the mean from 3 independent experiments ± SEM.

Treatment resistance remains a major problem in oncology. We therefore evaluated whether COTI-2 induces resistance in cancer cells and compared it to paclitaxel and cisplatin. DMS-153 and SHP-77 SCLC and A549 non-small cell lung cancer (NSCLC) cell lines were grown for 5 successive generations during exposure to COTI-2, cisplatin, or paclitaxel. While tumor cells showed higher levels of acquired resistance to paclitaxel and cisplatin after each round of treatment, they remained sensitive to COTI-2 across multiple generations (Fig 5). These data suggest that unlike paclitaxel and cisplatin, COTI-2 did not induce acquired resistance in the cell lines examined.

### Chemo-resistant cancer cell lines show no or little cross-resistance to COTI-2

**Fig 6:**
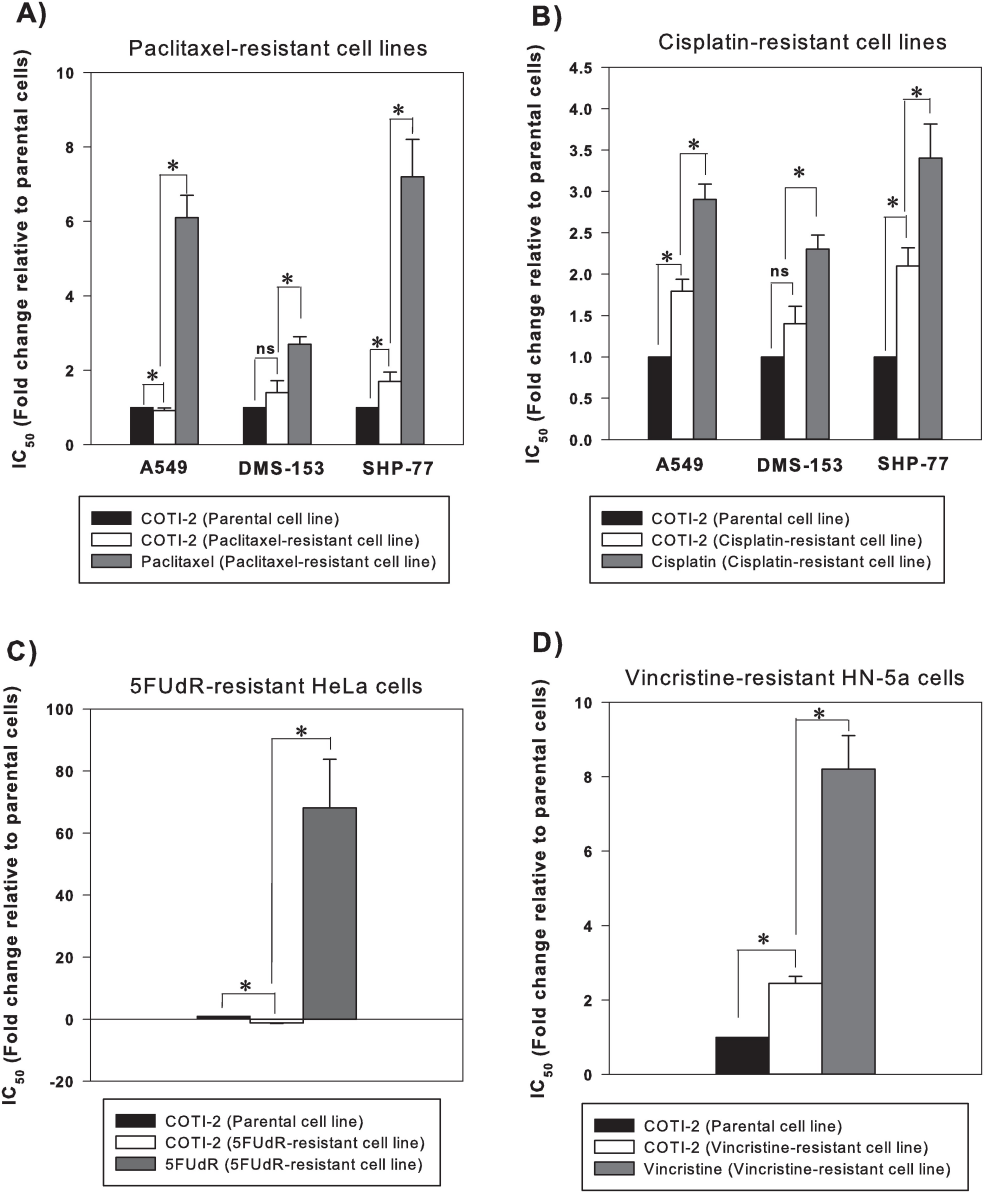
Chemo-resistant cancer cell lines often do not show cross-resistance to COTI-2. Paclitaxel-resistant (A) and cisplatin-resistant (B) A549, DMS-153, and SHP-77 cells were exposed to IC_50_ concentrations of COTI-2 and tumor cell proliferation was measured after approximately 4 doublings of control cells. 5FUdR-resistant HeLa cells (C) and vincristine-resistant HN-5a cells (D) were exposed to IC_50_ concentrations of COTI-2 as described in (A) and (B). Significant differences were assessed by Student’s *t*-test (*p*<0.05). Data indicates mean values derived from 3 independent experiments ± SEM.

Often cancer cells that develop acquired resistance to one chemotherapeutic agent also exhibit cross-resistance to other agents [18]. Although COTI-2 did not induce acquired resistance in human cancer cell lines after 4 rounds of selection, we determined whether cancer cells that acquired resistance to paclitaxel, cisplatin, 5FUdR, and vincristine would exhibit cross-resistance to COTI-2. The tested cell lines included cervical carcinoma HeLa cells resistant to 5-FUdR, HNSCC HN-5a cells resistant to vincristine, NSCLC A549 cells resistant to paclitaxel and cisplatin, and SCLC DMS-153 and SHP-77 cells resistant to paclitaxel and cisplatin. The parental cell line from which resistant lines were generated were used as non-resistant comparators. Paclitaxel-resistant A549 cells were more sensitive to COTI-2 than the parental cells (Fig 6A). Paclitaxel-resistant DMS-153 cells did not exhibit cross-resistance to COTI-2. Paclitaxel-resistant SHP-77 cells, however, did show minimal cross-resistance to COTI-2 but remained more sensitive to COTI-2 than paclitaxel. Cross-resistance to COTI-2 was evident to a minor degree in cisplatin-resistant A549 and SHP-77 cells but to a much lesser extent than resistance to cisplatin (Fig 6B). Cisplatin-resistant DMS-153 cells remained sensitive to COTI-2. The 5-FUdR-resistant HeLa cells were significantly more sensitive to COTI-2 (Fig 6C). Vincristine-resistant HN-5a cells exhibited cross-resistance to COTI-2 but to a much lesser degree than their resistance to vincristine (Fig 6D). Collectively, these data suggest that COTI-2 may be useful in salvage treatment after first-line treatment failure with common chemotherapy drugs. It is formally possible that lack of acquired- or cross-resistance to COTI-2 is due to COTI-2-mediated depletion of glutathione (GSH). Increased GSH and GGT levels in cancer cells and increased GSH activity linked to multi-drug resistance proteins (MRP) are associated with development of resistance to a wide variety of chemotherapeutic agents including cisplatin, 5FUdR, and paclitaxel [19, 20]. *N-*acetyl-L-cysteine (NAC) is a precursor in the glutathione synthesis pathway in mammalian cells and organs, and supplementation of human tumor cells with NAC leads to increased GSH and resistance to generators of toxic free radicals [21]. On the other hand, buthionine sulfoximine (BSO) reduces cellular GSH and sensitizes cells to free radicals [21]. We assessed the capacity of NAC and BSO treatment to alter the cytotoxicity of COTI-2 in DMS-53 cells. Addition of NAC or BSO did not affect the cytotoxic effect of COTI-2 (S4 Fig). Therefore, the potential effect of COTI-2 on GSH levels cannot explain the lack of acquired or cross-resistance to COTI-2.

## Discussion

We have previously shown that COTI-2, a third generation thiosemicarbazone, is active against a wide variety of human cancer cell lines with superior cytotoxic and anti-proliferative activity compared to multiple conventional chemotherapeutic and targeted agents [6]. The aim of this study was to evaluate the effect of COTI-2 in combination with standard therapeutic agents with different modes of action and to assess whether cancer cells develop acquired- and cross-resistance to COTI-2.

COTI-2 effects in combination with the two most clinically-used classes of tubulin-binding drugs, vinorelbine and paclitaxel, were evaluated. Both classes disrupt mitosis by interfering with microtubule assembly (the former destabilizing and the latter stabilizing microtubules) [22]. COTI-2 enhanced the cytotoxic activity of paclitaxel against SCLC cells in a synergistic manner (Fig 1A and 1C), suggesting the therapeutic efficacy of this combination. This data was further confirmed *in vivo* in an AN3-CA endometrial tumor model where the combination of COTI-2 and paclitaxel was not only synergistic but also demonstrated little toxicity (Fig 1E and S1 Fig). This data is promising since COTI-2 is currently in a phase I trial of gynaecologic malignancies and HNSCC (NCT02433626) and paclitaxel is commonly used to treat gynaecological malignancies [11, 12]. Unlike paclitaxel, vinorelbine was not synergistic with COTI-2 in the SCLC cells (Fig 2C and 2D). It is unclear why the two tubulin-binding drugs have a different effect when combined with COTI-2. The differences are likely due to the other mechanisms of paclitaxel and vinorelbine.

Cisplatin and its less toxic analogue carboplatin, both used in the treatment of various solid tumors including ovarian cancer [11, 12, 23], are intercalating agents that cause DNA intra-strand and inter-strand crosslinks [24]. Similar to paclitaxel, both cisplatin and carboplatin exhibited synergistic activity when combined with COTI-2 in SCLC cell lines (Figs 1C and 1D and 2A and 2B). The nature of interaction between these alkylating agents and COTI-2 appears to be a facilitating action since both cisplatin and carboplatin induce DNA damage and COTI-2 acts on mutant p53 [17] and induces apoptosis in response to DNA damage [6].

Gemcitabine, an antimetabolite or nucleoside analogue that inhibits DNA synthesis, and abraxane, which is paclitaxel protein-bound particles, are both used to treat pancreatic cancer [25, 26]. Combining COTI-2 and gemcitabine was not synergistic in SCLC cell lines (S3 Fig). The lack of synergy between gemcitabine and COTI-2 appears to be cell-specific because the combination of COTI-2 and gemcitabine was synergistic in PANC-1 xenografts (Fig 2E). COTI-2 plus abraxane significantly delayed PANC-1 xenograft growth in mice (Fig 2F). This combination showed early significant response to treatment compared to abraxane alone. This is important because early response to treatment correlates with improved survival in patients [27, 28]. We should note that we do not yet know whether the combination of COTI-2 with a cytotoxic agent *in vivo* results in improved tumor control due to increased apoptosis of tumor cells, increased cytostasis of tumor cells, a combination of the two, or other effects reducing overall tumor volume (for example, altered inflammatory activity, stromal cell viability/proliferation, angiogenesis, and the like). Future studies are warranted to clarify the COTI-2 mechanism of action *in vivo*.

In addition to chemotherapeutic agents, COTI-2 was evaluated in combination with the targeted-therapy agents, cetuximab, erlotinib, temsirolimus, and rapamycin/sirolimus. Combining COTI-2 with these agents *in vitro* also showed superior anti-proliferative effects compared to monotherapy. Previously, we demonstrated that COTI-2 was significantly more effective than erlotinib and cetuximab against human colorectal cancer cell lines *in vitro* [6]. When combined with these EGFR-targeting agents, COTI-2 was more effective at inhibiting tumor cell proliferation (Fig 4). Interestingly, COTI-2 was efficacious regardless of the EGFR and KRAS mutational status of these cell lines; the HCT-15 cell line carries an EGFR mutation conferring resistance to erlotinib whereas the COLO-205 and SW-620 cell lines carry a KRAS mutation conferring resistance to cetuximab [29]. Since the aforementioned mutations allow the activation of the RAS/MAPK pathway, we propose that the mechanism of action of COTI-2 does not appear to involve this pathway. In fact, ongoing mechanistic studies in our laboratory suggest that COTI-2 acts on mutant p53 and negatively modulates the PI3K/AKT/mTOR pathway [17].

Emergence of resistance is an ever-increasing problem in oncology and the use of combination therapies is rationalized as a means to potentially decrease resistance in cancer cells [1]. Furthermore, evaluating cross-resistance patterns is important since intrinsic cross-resistance may hamper the development of combination therapies. Since acquired- and cross-resistance to paclitaxel and platinum agents are common [30], COTI-2 was evaluated for cross-resistance to these agents. COTI-2 exhibited no or little cross-resistance to cisplatin- and paclitaxel-resistant cell lines in a cell-line specific manner (Fig 6A and 6B). In fact, collateral sensitivity to COTI-2 was observed in the A549 paclitaxel-resistant cell line suggesting, importantly, that resistance to paclitaxel sensitized the cells to COTI-2. Furthermore, unlike paclitaxel and cisplatin, COTI-2 did not induce acquired resistance in cancer cell lines following 4 rounds of selection (Fig 5). This suggests that COTI-2 can be valuable in second- or third-line treatment of chemotherapy-refractory tumors and that its mechanism of action is distinct from that of paclitaxel and cisplatin.

COTI-2 was also evaluated for cross-resistance to vincristine- and 5-FUdR-resistant cell lines. Vincristine-resistant cells were resistant to COTI-2 although to a much lesser degree than vincristine (Fig 6D) whereas COTI-2 was effective against cells resistant to 5-FUdR (Fig 6C). Similar to the paclitaxel-resistant A549 cells, the 5-FUdR-resistant HeLa cells also demonstrated collateral sensitivity to COTI-2. It is unclear why cross-resistance to COTI-2, although to a minor degree, develops to certain DNA-damaging agents but not to others. Further pre-clinical and clinical studies are warranted. The lack of acquired- or cross-resistance to COTI-2 was not related to COTI-2-mediated depletion of GSH in DMS-53 cells. However, there is always the possibility that other cell lines react differently to treatment with NAC and BSO. Therefore, future studies with a broad range of cell lines treated with NAC and BSO (*in vitro* and *in vivo*) and search for other underlying molecular mechanisms are warranted to shed light on this phenomenon.

In conclusion, we have shown that combining COTI-2 with commonly used therapeutic agents improves their efficacy against human cancer cell lines *in vitro* and *in vivo*. Importantly, the enhanced treatment effects appear to be most noticeable when COTI-2 is combined with taxanes and platinums. The combination treatment regimens tested did not result in any overt signs of toxicity *in vivo*. Furthermore, cancer cells do not develop resistance to COTI-2 after repeated rounds of drug selection unlike agents such as paclitaxel and cisplatin. Finally, cancer cells resistant to multiple chemotherapeutic drugs showed little or no cross-resistance to COTI-2.

## Supporting information

S1 Fig COTI-2 in combination with paclitaxel does not induce severe weight loss in animals. AN3-CA human endometrial cells (1 x 10^7^) were injected into the right flanks of athymic nude mice (n = 10 mice per group). Xenografts were grown to an average volume of 170 mm^3^ before animals received treatment i.v.. Vehicle control and COTI-2 (25 mg/kg) were administered 3 times a week on alternate days until study end. The schedule for paclitaxel was daily for 5 days (5 mg/kg). In the combination arm, animals received COTI-2 (25 mg/kg) 3 times a week on alternate days until study end and 5 daily injections of paclitaxel (5 mg/kg). Animals in the COTI-2 monotherapy group exhibited a maximum weight loss of 4.7% on day 6, which was recovered later. With paclitaxel monotherapy a maximum weight loss of 8.0% was noted, however the weight was recovered by day 17. Animals in the combination arm exhibited a moderate weight loss of 10.8% on day 6 of the study, which was recovered later.
(TIF)

S2 Fig Combining treatment of COTI-2 and carboplatin is more effective in delaying OVCAR-3 xenograft growth than either drug alone. OVCAR-3 human ovarian carcinoma cells (5 X 10^5^ cells) were injected into each flank of NIH III nu/nu mice (4–8 weeks old) (n = 6 mice per group). Xenografts were grown to ~100 mm^3^ before animals received treatment, which consisted of the vehicle control, COTI-2 (30 mg/kg), carboplatin (25 mg/kg), or the combination (COTI-2 at 30 mg/kg and carboplatin at 25 mg/kg). COTI-2 was delivered *p*.*o*. with a schedule of 5 days on treatment and 2 days off weekly starting on day one. Carboplatin was administered i.p. as a single dose on day one. The dosing schedule for the combination treatments was identical to that of the single agent treatments for each drug. COTI-2 administration was initiated 1 day after treatment with either gemcitabine or abraxane. *Significantly different from single agent carboplatin group, Student’s *t*-test, *p*<0.05.
(TIF)

S3 Fig Combining COTI-2 with gemcitabine does not enhance activity against human SCLC cells. DMS-114 (A) and SHP-77 (B) SCLC cells were treated with various concentrations of gemcitabine in combination with or without an IC_25_ concentration of COTI-2 for 4 days before cell viability was determined. There was no significant difference (*p*<0.05, Student’s *t*-test) between monotherapy and the combination treatment. Data are the average mean of 3 independent experiments ± SEM.
(TIF)

S4 Fig Effect of COTI-2 and NAC or BSO treatment on DMS-53 tumor cell proliferation. Cell viability was determined using the alamarBlue assay following COTI-2 or COTI-2 plus NAC (A) or BSO (B) exposure for 4 days. Data points represent the mean of 5 independent replicates.
(TIF)

S1 Table Mortality in mice treated with the vehicle control, carboplatin, and combinations of COTI-2 and carboplatin. (PDF)
