# Supplementary figures and images for: Novel anti-cancer drug COTI-2 synergizes with therapeutic agents and does not induce resistance or exhibit cross-resistance in human cancer cell lines
Source: PLoS One. 2018 Jan 24;13(1):e0191766. doi: 10.1371/journal.pone.0191766 (PMC5783418; doi:10.1371/journal.pone.0191766)

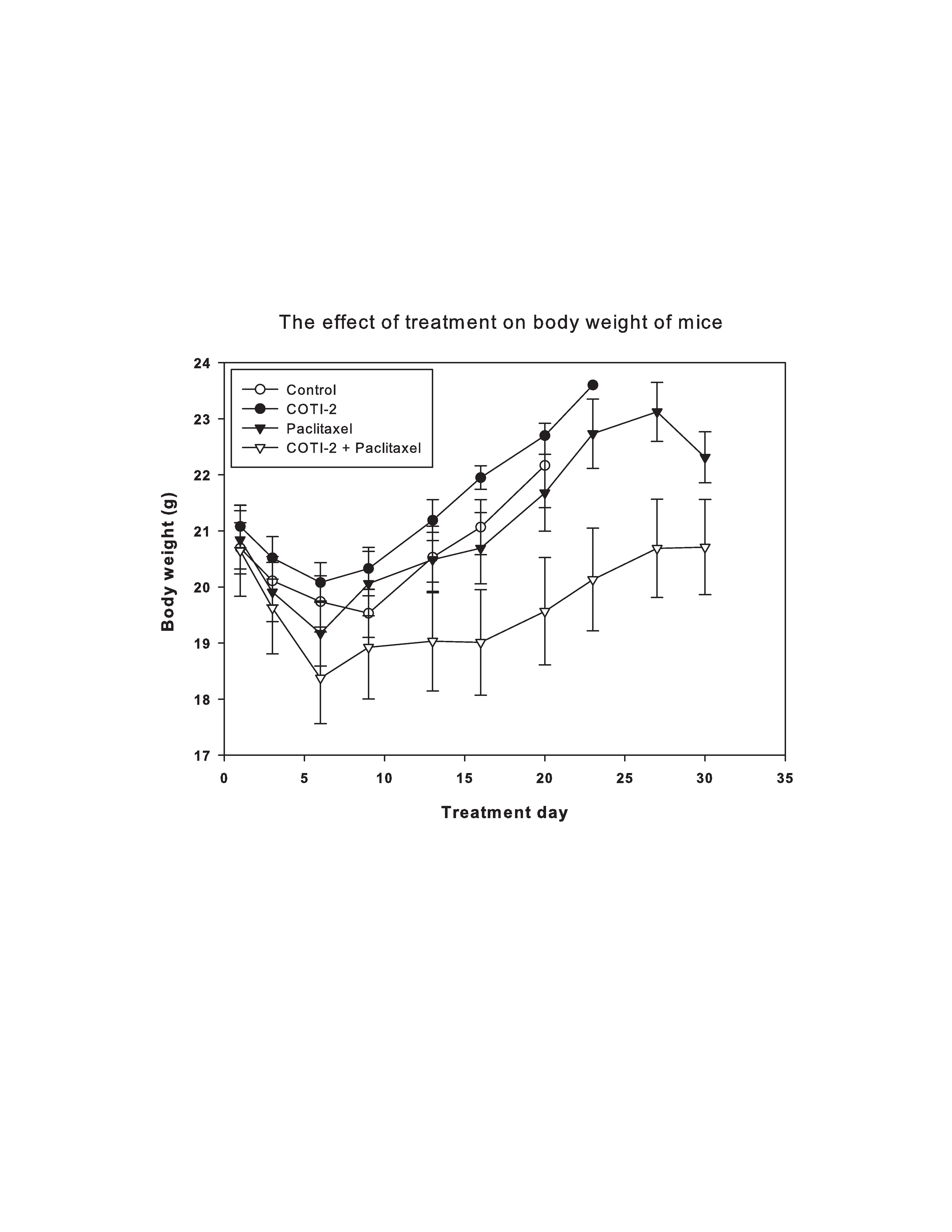

Supplement: S1 Fig — AN3-CA human endometrial cells (1 x 10^7^) were injected into the right flanks of athymic nude mice (n = 10 mice per group). Xenografts were grown to an average volume of 170 mm3 before animals received treatment i.v.. Vehicle control and COTI-2 (25 mg/kg) were administered 3 times a week on alternate days until study end. The schedule for paclitaxel was daily for 5 days (5 mg/kg). In the combination arm, animals received COTI-2 (25 mg/kg) 3 times a week on alternate days until study end and 5 daily injections of paclitaxel (5 mg/kg). Animals in the COTI-2 monotherapy group exhibited a maximum weight loss of 4.7% on day 6, which was recovered later. With paclitaxel monotherapy a maximum weight loss of 8.0% was noted, however the weight was recovered by day 17. Animals in the combination arm exhibited a moderate weight loss of 10.8% on day 6 of the study, which was recovered later. (TIF) [file pone.0191766.s001.tif]

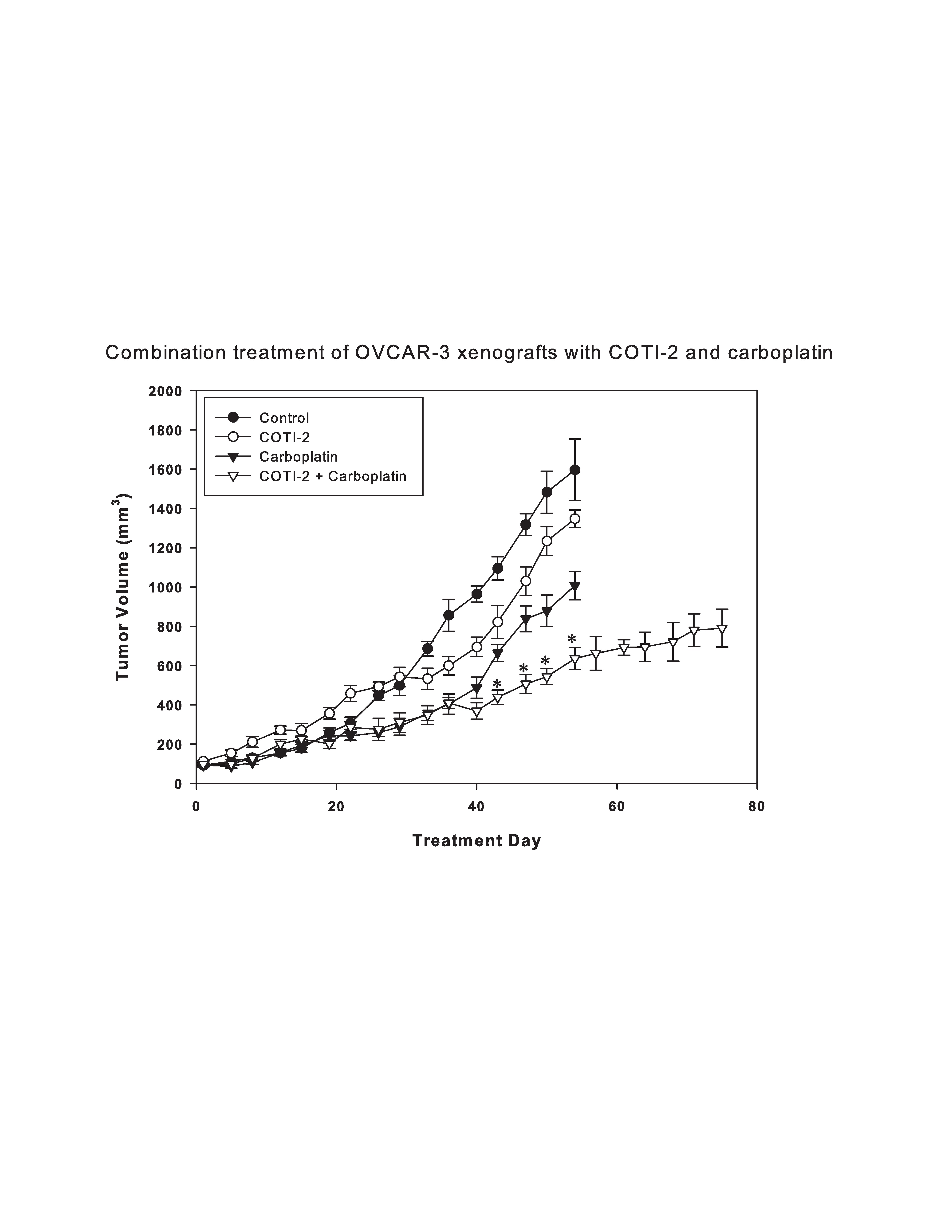

Supplement: S2 Fig — OVCAR-3 human ovarian carcinoma cells (5 X 105 cells) were injected into each flank of NIH III nu/nu mice (4–8 weeks old) (n = 6 mice per group). Xenografts were grown to ~100 mm3 before animals received treatment, which consisted of the vehicle control, COTI-2 (30 mg/kg), carboplatin (25 mg/kg), or the combination (COTI-2 at 30 mg/kg and carboplatin at 25 mg/kg). COTI-2 was delivered p.o. with a schedule of 5 days on treatment and 2 days off weekly starting on day one. Carboplatin was administered i.p. as a single dose on day one. The dosing schedule for the combination treatments was identical to that of the single agent treatments for each drug. COTI-2 administration was initiated 1 day after treatment with either gemcitabine or abraxane. *Significantly different from single agent carboplatin group, Student’s t-test, p<0.05. (TIF) [file pone.0191766.s002.tif]

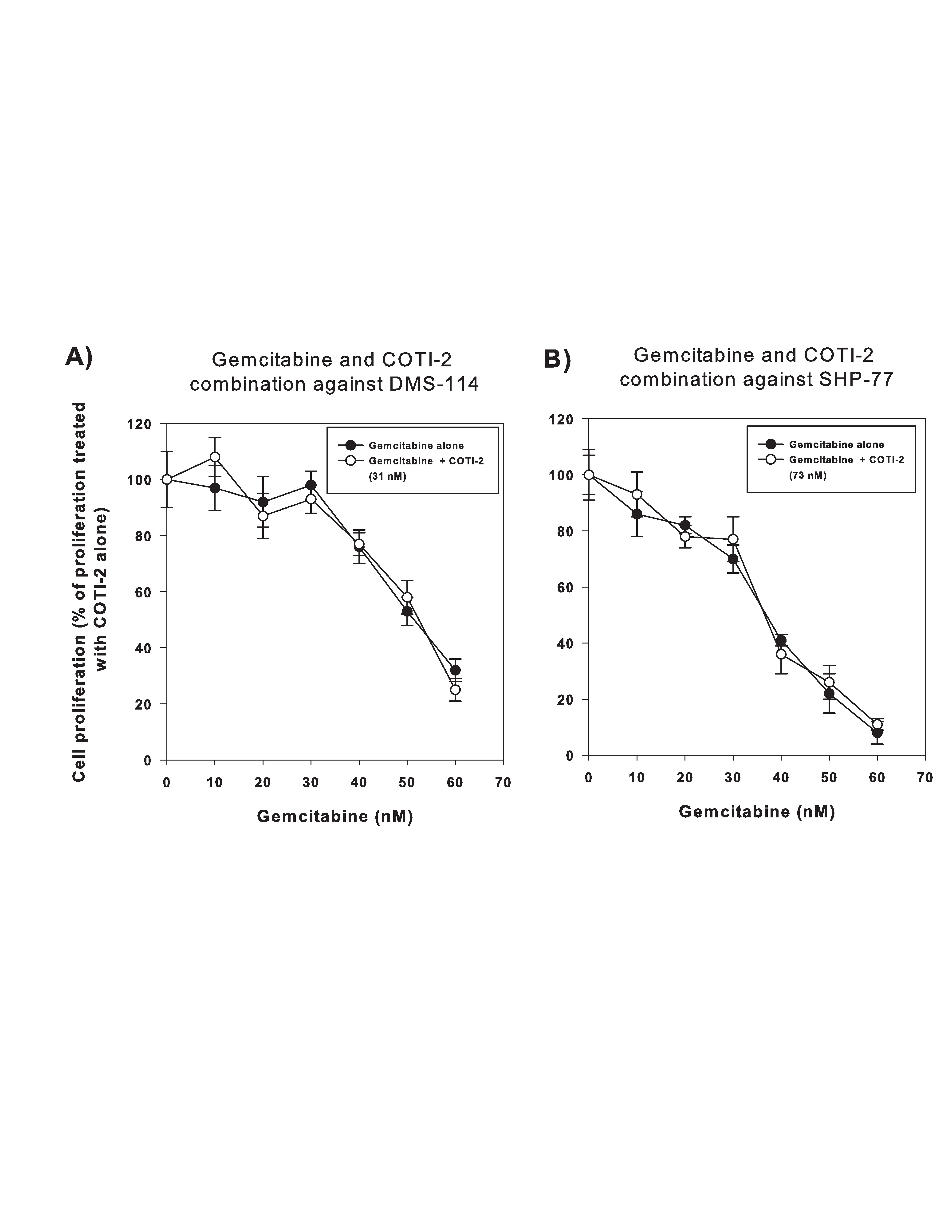

Supplement: S3 Fig — DMS-114 (A) and SHP-77 (B) SCLC cells were treated with various concentrations of gemcitabine in combination with or without an IC25 concentration of COTI-2 for 4 days before cell viability was determined. There was no significant difference (p<0.05, Student’s t-test) between monotherapy and the combination treatment. Data are the average mean of 3 independent experiments ± SEM. (TIF) [file pone.0191766.s003.tif]

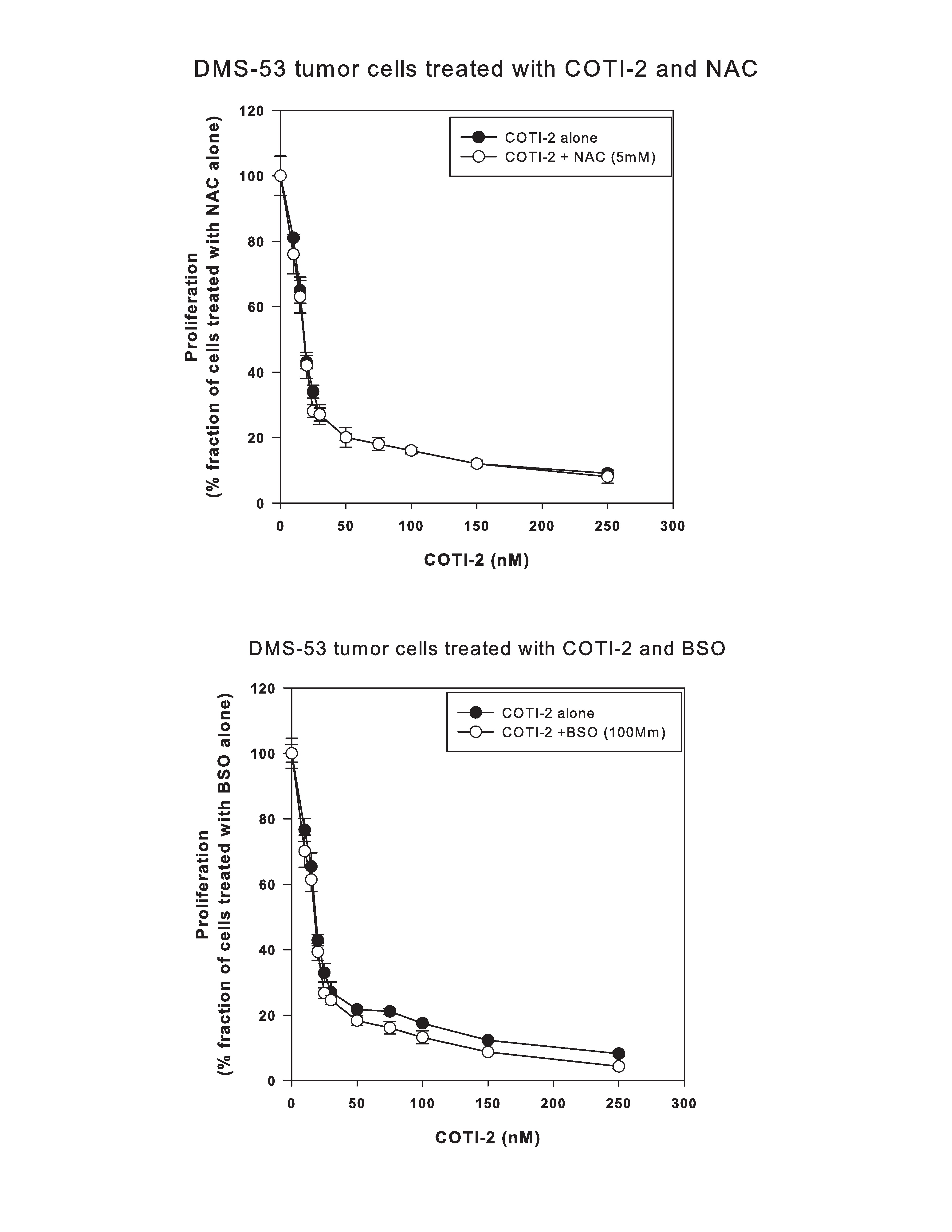

Supplement: S4 Fig — Cell viability was determined using the alamarBlue assay following COTI-2 or COTI-2 plus NAC (A) or BSO (B) exposure for 4 days. Data points represent the mean of 5 independent replicates. (TIF) [file pone.0191766.s004.tif]
