# Supplementary material for: Novel anti-cancer drug COTI-2 synergizes with therapeutic agents and does not induce resistance or exhibit cross-resistance in human cancer cell lines
Source: PLoS One. 2018 Jan 24;13(1):e0191766. doi: 10.1371/journal.pone.0191766 (PMC5783418; doi:10.1371/journal.pone.0191766)
Supplement: S1 Table — (PDF) [file pone.0191766.s005.pdf]

S1_Table

		Number of surviving animals alive/number of treated animals (% survival)					
**Day**	**Vehicle Control**	**COTI-2 alone (30 mg/kg)**	**Carboplatin alone (25 mg/kg)**	**Carboplatin alone (35 mg/kg)**	**Carboplatin alone (50 mg/kg)**	**COTI-2 (30 mg/kg) + Carboplatin (25 mg/kg)**	**COTI-2 (30 mg/kg) + Carboplatin (50 mg/kg)**
1	6/6 (100%)	(30 mg/kg)	6/6 (100%)	6/6 (100%)	6/6 (100%)	6/6 (100%)	6/6 (100%)
5	6/6 (100%)	6/6 (100%)	6/6 (100%)	6/6 (100%)	5/6 (83%)	6/6 (100%)	2/6 (33%)
8	6/6 (100%)	6/6 (100%)	6/6 (100%)	5/6 (83%)	4/6 (67%)	6/6 (100%)	0/6 (0%)
12	6/6 (100%)	6/6 (100%)	6/6 (100%)	5/6 (83%)	2/6 (33%)	6/6 (100%)	0/6 (0%)
19	6/6 (100%)	6/6 (100%)	6/6 (100%)	4/6 (67%)	1/6 (17%)	6/6 (100%)	0/6 (0%)
22	6/6 (100%)	6/6 (100%)	6/6 (100%)	2/6 (33%)	0/6 (0%)	6/6 (100%)	0/6 (0%)
26	6/6 (100%)	6/6 (100%)	6/6 (100%)	2/6 (33%)	0/6 (0%)	6/6 (100%)	0/6 (0%)
29	6/6 (100%)	6/6 (100%)	6/6 (100%)	2/6 (33%)	0/6 (0%)	6/6 (100%)	0/6 (0%)
33	6/6 (100%)	6/6 (100%)	6/6 (100%)	2/6 (33%)	0/6 (0%)	6/6 (100%)	0/6 (0%)
36	6/6 (100%)	6/6 (100%)	6/6 (100%)	2/6 (33%)	0/6 (0%)	6/6 (100%)	0/6 (0%)
40	6/6 (100%)	6/6 (100%)	6/6 (100%)	2/6 (33%)	0/6 (0%)	6/6 (100%)	0/6 (0%)
43	6/6 (100%)	6/6 (100%)	6/6 (100%)	2/6 (33%)	0/6 (0%)	6/6 (100%)	0/6 (0%)
47	6/6 (100%)	6/6 (100%)	6/6 (100%)	2/6 (33%)	0/6 (0%)	6/6 (100%)	0/6 (0%)
50	6/6 (100%)	6/6 (100%)	6/6 (100%)	2/6 (33%)	0/6 (0%)	6/6 (100%)	0/6 (0%)
